# Supplementary material for: A Hypothalamic Mechanism Regulates the Duration of a Migraine Attack: Insights from Microstructural and Temporal Complexity of Cortical Functional Networks Analysis
Source: Int J Mol Sci. 2022 Oct 31;23(21):13238. doi: 10.3390/ijms232113238 (PMC9658908; doi:10.3390/ijms232113238)
Supplement: Supplementary file 1 [file ijms-23-13238-s001.zip › ijms-1936025-supplementary.pdf]

Network	***Independent Component, correlation index***		
Cerebellum	*IC1*<br>*0.36*		
Salience	*IC10, IC19*<br>*0.32, 0.25*		
Auditory	*IC2*<br>*0.38*		
Dorsal attention	*IC6, IC17*<br>*0.21, 0.24*		
Default Mode	*IC27, IC28, IC31*<br>*0.10, 0.64, 0.19*	*IC25, IC21*<br>*0.17, 0.17*	*IC20, IC15*<br>*0.29, 0.25*
Frontal parietal	*IC3, IC23*<br>*0.31, 0.32*		
Language	*IC26*<br>*0.21*		
Sensory motor	*IC33, IC7*<br>*0.21, 0.19*	*IC8*<br>*0.14*	
Visual	*IC24*<br>*0.36*	*IC11*<br>*0.22*	

Supplementary Table S1*: Correlation's values for each network previously identified with reference to the networks' templates*
